# Association between Serum Mg^2+^ Concentrations and Cardiovascular Organ Damage in a Cohort of Adult Subjects

**DOI:** 10.3390/nu12051264

**Published:** 2020-04-29

**Authors:** Elettra Mancuso, Maria Perticone, Rosangela Spiga, Carolina Averta, Mariangela Rubino, Teresa Vanessa Fiorentino, Sofia Miceli, Gaia Chiara Mannino, Angela Sciacqua, Elena Succurro, Francesco Perticone, Giorgio Sesti, Francesco Andreozzi

**Affiliations:** 1 Department of Medical and Surgical Sciences, University Magna Graecia of Catanzaro, 88100 Catanzaro, Italy; elettramancuso@gmail.com (E.M.); mariaperticone@hotmail.com (M.P.); rosangela.spiga@gmail.com (R.S.); carolinaaverta90@gmail.com (C.A.); marubino83@gmail.com (M.R.); vanessa.fiorentino@unicz.it (T.V.F.); sofy.miceli@libero.it (S.M.); sciacqua@unicz.it (A.S.); succurro@unicz.it (E.S.); perticone@unicz.it (F.P.); andreozzif@unicz.it (F.A.); 2 Department of Clinical and Molecular Medicine, University “Sapienza” of Rome, 00189 Rome, Italy; giorgio.sesti@uniroma1.it

**Keywords:** magnesium, subclinical atherosclerosis, atherosclerosis, cardiovascular disease, carotid-intima media thickness, left ventricular mass index

## Abstract

Magnesium (Mg^2+^) levels are associated with insulin resistance, hypertension, atherosclerosis, and type 2 diabetes (T2DM). We evaluated the clinical utility of physiological Mg^2+^ in assessing subclinical cardiovascular organ damage including increased carotid artery intima- media thickness (c-IMT) and left ventricular mass index (LVMI) in a cohort of well-characterized adult non-diabetic individuals. Age- and gender-adjusted correlations between Mg^2+^ and metabolic parameters showed that Mg^2+^ circulating levels were correlated negatively with body mass index (BMI), fasting glucose, and 2h-oral glucose tolerance test (OGTT) glucose. Similarly, Mg^2+^ levels were significantly and negatively related to c-IMT and LVMI. A multivariate regression analysis revealed that age (β = 0.440; *p* < 0.0001), BMI (β = 0.225; *p* < 0.0001), and Mg^2+^ concentration (β = −0.122; *p* < 0.01) were independently associated with c-IMT. Age (β = 0.244; *p* = 0.012), Mg^2+^ (β = −0.177; *p* = 0.019), and diastolic blood pressure (β = 0.184; *p* = 0.038) were significantly associated with LVMI in women, while age (β = 0.211; *p* = 0.019), Mg^2+^ (β = −0.171; *p* = 0.038) and the homeostasis model assessment index of insulin resistance (HOMA-IR) (β = −0.211; *p* = 0.041) were the sole variables associated with LVMI in men. In conclusion, our data support the hypothesis that the assessment of Mg^2+^ as part of the initial work-up might help unravel the presence of subclinical organ damage in subjects at increased risk of cardiovascular complications.

## 1. Introduction

Magnesium (Mg^2+^), the principal intracellular divalent cation, is vital for a healthy human body and its importance has been highlighted since 1933, when Kruse et al. [1] reported the negative effects of acute Mg^2+^ deficiency in rats. Serum concentrations of Mg^2+^ is not routinely included as part of the automated chemistry profile and hypomagnesemia has been commonly unrecognized [2]. For this reason, Mg^2+^ has been defined as the “forgotten cation” in clinical practice [3]. Indeed it is nowadays recognized that Mg^2+^ plays a key role in a wide range of fundamental cellular reactions and biological processes, such as DNA synthesis, RNA expression [4], protein synthesis, and the regulation and catalysis of many enzymatic reactions [5]. At a molecular level, it forms a crucial complex with adenosine triphosphate and neutralizes its negative charge to facilitate binding to enzymes [6], and it activates rate-limiting glycolytic and tricarboxylic acid cycle enzymes regulating intermediary metabolism [7]. Furthermore, it is involved in fundamental physiological mechanisms for transfer, storage, and use of energy [8,9].

Clinical and experimental evidences suggest that serum Mg^2+^ concentration decreases in humans and animals in several chronic diseases [10]. Values below the threshold of 0.5 mM [11] are indicative of hypomagnesemia [12] and could result from redistribution of Mg^2+^ from the extracellular to the intracellular space, reduced intake, intestinal absorption alterations, and gastrointestinal loss [11]. 

It has been demonstrated that Mg^2+^ has vasodilatory, anti-inflammatory, anti-ischemic, and antiarrhythmic properties; thus, it is presumably a useful therapeutic agent in cardiovascular medicine. Indeed, several epidemiological studies have established that hypomagnesemia may increase the risk of cardiovascular disease (CVD) [13]. Accordingly, randomized controlled trials and meta-analyses have revealed an inverse association between dietary Mg^2+^ intake and a large number of preclinical and clinical risk factors of CVD [14], hypertension, atherosclerosis, stroke, cardiac arrhythmias [15], abnormal lipid metabolism, insulin resistance, metabolic syndrome, and type 2 diabetes mellitus (T2DM) [16].

The task of assessing subclinical organ damage is a crucial key in the identification of subjects with primary hypertension. In fact, a diagnosis of left ventricular hypertrophy (LVH) or peripheral atherosclerosis strongly increases the overall cardiovascular risk profile and may be helpful for deciding whether to begin treatment or to detect optimal target blood pressure [17]. Carotid artery intima-media thickness (c-IMT) is a well-established subclinical marker of vascular damage, and it has been shown to predict cardiovascular events [18,19,20]. Increased left ventricular mass index (LVMI) is another independent organ damage index that has been associated with cardiovascular morbidity and mortality in the general population [21].

In this study, we aimed to analyze the clinical utility of physiological Mg^2+^ levels in assessing subclinical cardiovascular organ damage, including increased carotid artery c-IMT and LVMI in a cohort of well-characterized adult non-diabetic individuals participating in the CATAnzaro MEtabolic RIsk factors (CATAMERI) study.

## 2. Materials and Methods

### 2.1. Study Population

The study population consisted of 413 unrelated Caucasian subjects (180 men and 233 women; mean age 44 ± 12 years), who were enrolled in the CATAMERIS, an observational study dedicated to the identification and characterization of cardio-metabolic risk factors [22,23]. The exclusion criteria were the presence of autoimmune diabetes, T2DM, chronic gastrointestinal diseases, chronic pancreatitis, a history of any malignant disease, a history of alcohol or drug abuse, and liver or kidney failure.

For all participants, anthropometrical parameters such as body mass index (BMI), waist circumference, systolic (SBP) and diastolic (DBP) blood pressure after a 12-h fast, were assessed, and blood samples were collected for biochemical measurements. Height was measured to the nearest 0.1 cm, while body weight was measured with a calibrated electronic scale to the nearest 0.1 kg. BMI was calculated as body weight in kilograms divided by height in square meters (kg/m^2^). A 75-g oral glucose tolerance test (OGTT) was performed with 0, 30, 60, 90, and 120 min sampling for circulating plasma glucose and insulin measurements.

The study was approved by the Institutional Ethics Committee of the University “Magna Graecia” of Catanzaro (approval code: 2012.63). Written informed consent was obtained from each subject in accordance with the principles of the Declaration of Helsinki.

### 2.2. Ultrasound Measurement of c-IMT and Echocardiographic Assessments

High resolution B-mode ultrasound was used to measure c-IMT of the common carotid artery using an ATL HDI 3000 ultrasound system (Advanced Technology Laboratories, Bothell, WA) equipped with a 7.5 MHz transducer, as previously described in [24]. Echocardiographic assessments were performed by a single experienced examiner, who was blinded to the clinical and laboratory results of the study group. Tracings were taken with patients in a partial left decubitus position using a VIVID-7 Pro ultrasound machine (GE Technologies, Milwaukee, WI) with an annular phased array 2.5-MHz transducer. Only frames with optimal visualization of cardiac structures were considered for reading. LVM was calculated using the Devereux equation [25] and normalized by body surface area (LVM index [LVMI]).

### 2.3. Laboratory Determinations

Blood levels of glucose, triglycerides, total cholesterol, and high-density lipoprotein (HDL) cholesterol levels were measured by enzymatic methods (Roche, Basel, Switzerland). HbA1c was assessed with high performance liquid chromatography using a National Glycohemoglobin Standardization Program (NGSP) certified automated analyzer (Adams HA-8160 HbA1C analyzer, Menarini, Italy). A chemiluminescence-based assay (Immulite^®^, Siemens, Italy) was used to measure serum insulin concentrations. Serum Mg^2+^ concentrations were measured by COBAS INTEGRA Mg^2+^, based on a colorimetric method assay (Roche Diagnostic, Mannheim, Germany).

### 2.4. Calculations

The homeostasis model assessment index of insulin resistance (HOMA-IR) was calculated as fasting insulin × fasting glucose/22.5 [26]. A value of c-IMT > 0.9 mm was used as the index of vascular atherosclerosis according to the 2018 ESC/ESH Guidelines for the management of arterial hypertension: The Task Force for the management of arterial hypertension of the European Society of Cardiology (ESC) and the European Society of Hypertension (ESH) [27]. The partition values adopted for the definition of hypertension-mediated organ damage by echocardiography were LVMI > 115 (men) and > 95 (women), as recommended by the 2018 ESC/ESH guidelines [27].

### 2.5. Statistical Analysis

Variables with skewed distribution (i.e., triglycerides, fasting insulin, and HOMA-IR) were log transformed to meet the normality assumption for statistical purposes. The results for continuous variables are given as means ± SD, whereas categorical variables are reported as percentages. The χ^2^ test was used for comparison of categorical variables within quartile groups. Correlation coefficients were calculated according to Pearson’s method. Anthropometric and cardio-metabolic variables were tested after adjusting for age, gender, and BMI using a general linear model with post hoc Bonferroni correction for multiple comparisons. A multivariable linear regression analysis was performed in order to evaluate the independent contribution of Mg^2+^ to c-IMT and LVMI. A logistic regression analysis adjusted for confounders was used to determine the strength of the association between the study groups and vascular atherosclerosis (c-IMT > 0.9 mm) [28] or hypertension-mediated organ damage (LVMI > 115 in males, LVMI > 95 in women) [27]. All analyses were performed using the statistical package SPSS 22.0 for Windows (SPSS, Chicago, IL, USA) and *p* ≤ 0.05 was considered statistically significant.

## 3. Results

The anthropometric and metabolic characteristics of the study group are summarized in Table 1. Notably, the study cohort was relatively young (44 ± 12 years), and it showed a wide range of BMI (30.6 ± 7.2) with a ~43.6% prevalence of obese subjects. Women were significantly younger than men, and they also showed significantly lower values of blood pressure, 2h-OGTT glucose levels, triglycerides, and LVMI. When comparing gender groups (men and women) no significant differences were observed in BMI, total cholesterol, fasting insulin, and HOMA-IR, while we observed significant variation in lipid profiles, 2h-OGTT glucose levels, and smoking habits. 

As shown in Table 1, men and women had similar distributions of c-IMT, whereas the mean value of LVMI differed significantly in the two groups (*p* < 0.0001).

The results of univariate correlations between Mg^2+^ concentration and anthropometric and cardio-metabolic variables in the whole study group are presented in Table 2. Mg^2+^ circulating levels were positively correlated with SBP (r = 0.105; *p* = 0.03) whereas were negatively correlated with BMI (r = −0.144; *p* < 0.01), 2h OGTT glucose (r = −0.108; *p* < 0.03), HOMA-IR (r = −0.102; *p* = 0.04), c-IMT (r = −0.113; *p* < 0.03) and LVMI (r = −0.122; *p* < 0.03). In a Pearson’s correlation analysis adjusted for age and gender, BMI (r = −0.176; *p* < 0.001), fasting glucose (r = −126; *p* < 0.03), 2h OGTT glucose (r = −0.164; *p* < 0.01), c-IMT (r = −0.140; *p* = 0.01) and LVMI (r = −0.209; *p* < 0.001) remained negatively correlated to Mg^2+^ circulating levels, and in addition HDL-Col showed a significant positive correlation (r = 0.124; *p* < 0.03).

To evaluate the independent factors influencing the variability of c-IMT, a multivariate linear stepwise regression analysis was run in a model including age, BMI, Mg^2+^, gender, SBP, DBP, total HDL- and LDL-cholesterol, HOMA-IR, and smoking habits. The three variables that remained and significantly associated with c-IMT were age (β = 0.440; *p* < 0.0001), BMI (β = 0.225; *p* < 0.0001), and Mg^2+^ concentration (β = −0.122; *p* < 0.01), accounting for 27.8 % of c-IMT variation (Table 3).

Next, a multiple linear regression analysis was separately performed in women and men in order to estimate the strength of the association between Mg^2+^ and LVMI (Table 4A,B, respectively). Comparison of standardized coefficients were allowed to determine the relative impact of each factors on LVMI. Age (β = 0.244; *p* = 0.012), Mg^2+^ (β = −0.177; *p* = 0.019) and DBP (β = 0.184; *p* = 0.038) were significantly associated with LVMI in women (Model 1), while when the multivariate regression analysis was performed in men (Model 2), age (β = 0.211; *p* = 0.019), Mg^2+^ (β = −0.171; *p* = 0.038), and HOMA-IR (β = −0.211; *p* = 0.041) were the sole statistically significant variables. Altogether, our models explained 21.6% of LVMI variance in women and 15.4% in men.

## 4. Discussion

Mg^2+^ has a pivotal role in the homeostasis of the organism because it is a cofactor of several enzymes involved in most cellular processes [29]. Alterations of Mg^2+^ levels have been involved in several pathologic conditions [30], and a tight regulation of Mg^2+^ absorption and excretion is fundamental for the well-being of the organism [31]. Intestinal Mg^2+^ uptake is counterbalanced by renal Mg^2+^ depletion in urine, and in the case of deficiency, circulating Mg^2+^ levels are maintained stable by deploying the bone or muscle reservoir [32].

Mg^2+^ has profusely been associated with several aspects of glucose metabolism, such as insulin resistance [33], which is understandable because many enzymes required for the control of metabolic pathways and for signaling transduction require Mg^2+^. Moreover, many authors have demonstrated a clear relationship between hypomagnesemia and cardio-metabolic events both in humans and in experimental animals [34,35]. In model rats supplemented with Mg^2+^ the onset of diabetes appeared to be delayed, while animals with low Mg^2+^ levels showed increased serum glucose levels and an alteration of their lipid pattern, thought to justify the association with incidental CVD [36,37,38], hypertension [39,40], or T2DM [41,42]. In addition to this, Mg^2+^ is also known as a physiological regulator of vascular tone with the ability of modulating contractile proteins and reducing peripheral vascular resistance [43]. Although its importance for the regulation of blood pressure and vascular tone [12] is widely accepted, Mg^2+^ role in the pathophysiology of CVD is still controversial [31,44,45], and very recently, an accurate meta-analysis of prospective cohort studies [46] discussed the difficulties of interpreting associations of Mg^2+^ levels intended as a quantitative variable. Our study supplies evidences of this complex phenomenon producing data obtained from a large population study.

Increased c-IMT is strongly associated with the risk of CV events, and it is considered a marker of preclinical atherosclerosis and a surrogate marker of structural remodeling of the arterial wall. It is often used as a predictor of acute coronary events [47,48] and severity of atherosclerosis [49,50,51,52,53]. Similarly, LVMI is considered a marker of preclinical and subclinical development of atherosclerosis and an estimate of vascular damage, and it is also a major determinant of CV mortality because of the contractile impairment of CV dysfunction [54,55].

Thus, to determine whether Mg^2+^ is independently associated with the early manifestation of atherosclerosis, we measured c-IMT by ultrasonography. We also evaluated the correlation between Mg^2+^ and LVMI, and we observed that Mg^2+^ levels were negatively correlated with both c-IMT and LVMI, further confirming that reduced Mg^2+^ increases CV risk. In addition, we applied a multiple regression analysis to evaluate the independent influence of Mg^2+^ on c-IMT and LVMI.

Our multivariate model included the whole study cohort when focusing on c-IMT, and the covariates together explained 27.8% of the overall variation in c-IMT. Regarding LVMI, the average values of this marker are known to be different according to gender, as reported by the ESC/ESH Guidelines for the management of arterial hypertension [27]; therefore, the multivariate analysis was performed distinctly for men and women. Furthermore, the incidence of nondiabetic CVD is lower in premenopausal women and it has been hypothesized that estrogens protect women against atherosclerotic complications [56]. Although we considered menopause as a potential confounder, and we included “presence of menopause” among the covariates of Model 1, it is possible that the discrepancy observed in the resulting statistical model (Model 1 explained 21.6% of LVMI variation for women; Model 2 explained 15.4% for men) could be due to the influence of estrogens, in the sense that the male gender, which is already prone to the development of CVD and lacks the beneficial influence of estrogens, might resent less of a Mg^2+^ decrement than its counterpart.

Our findings are in accordance with previous evidences obtained in vitro and in clinical studies that have correlated lower Mg^2+^ levels with vascular calcification [57], cardiovascular mortality [58], and several markers of CVD [13,14,15].

The strength of our study is the sample size and the homogeneity of the study cohort, which minimizes differences in dietary habits and nutritional conditions. At the same time, the geographical and ethnical restriction of our cohort might represent a limitation to the generalizability of the data, since the results refer exclusively to Caucasian subjects from Southern Italy. Other solid aspects are exclusions of known confounding factors that could be associated with cardiovascular alterations, including metabolic disorders.

The current study has some weaknesses due to its observational nature. Indeed, making causal interpretations of associations between diagnostic parameters and the risk of CVD is hazardous when it is not accomplished in a longitudinal context. In spite of this issue, it should be noted that our results reflect only an association with early atherosclerosis and not incident CVD, which is consistent with the relative young age of our study subjects. Furthermore, our study cohort was recruited at a referral university hospital, representing subjects carrying at least one cardio-metabolic risk factor, it showed a ~43.6% prevalence of obesity, and therefore, the current findings may not be extendible to the healthy general population. Finally, serum Mg^2+^ concentrations and clinical parameters were measured only once, at the beginning of the study.

Hypomagnesaemia is a CV risk factor in the general population, and our study highlights a significant negative relationship between normal range of Mg^2+^ and CV risk markers, such as LVMI and c-IMT in a normal population. Although c-IMT and LVMI are widely used as validated indices of early atherosclerosis and vascular damage and as surrogate marker of CVD, further prospective studies with incident cases of CV events are necessary to confirm these findings.

Our evaluation of c-IMT and LVMI strongly suggests that measuring serum Mg^2+^ concentrations could be a useful and inexpensive instrument for the precocious identification of subgroups of hypertensive patients with asymptomatic subclinical vascular atherosclerotic disease and with higher cardiovascular risk. Furthermore, our data indicate that higher serum Mg^2+^ concentrations may play a key protective role in the development of vascular calcification and, in general, for atherovascular risks.

In conclusion, the present study confirms and extends previous clinical data on the role of Mg^2+^ as a marker of increased cardiovascular risk in hypertension, and we propose to include the assessment of magnesium as part of the initial work-up of hypertensive patients.

## Figures and Tables

**Table 1:** Anthropometric and metabolic characteristics of the study subjects.

Variables	Whole Study Group	Male	Female	*p*
Gender (M/F)	413	180	233	<0.01 *
Age (years)	44 (±12)	46 (±11)	43 (±12)	<0.03 **
BMI (Kg/m^2^)	30.6 (±7.2)	30.2 (±5.5)	30.9 (±8.2)	0.305 ***
SBP (mmHg)	123.2 (±15.0)	128.9 (±13.7)	119.0 (±14.5)	<0.0001
DBP (mmHg)	77.6 (±10.2)	80.7 (±9.4)	75.2 (±10.2)	<0.0001
Total cholesterol (mg/dl)	197.9 (±35.7)	198.7 (±34.1)	197.2 (±36.8)	0.67
HDL-Chol (mg/dl)	50.9 (±13.9)	43.1 (±10.1)	56.8 (±13.5)	<0.0001
LDL-Chol (mg/dl)	126.0 (±30.8)	129.4 (±30.1)	123.3 (±31.1)	<0.05
Triglycerides (mg/dl)	122.8 (±71.7)	139.9 (±76.3)	109.6 (±64.9)	<0.0001
Mg^2+^ (mg/dl)	2.00 (±0.16)	2.02 (±0.16)	1.98 (±0.16)	<0.05
Fasting glucose (mg/dl)	92.8 (±10.5)	94.9 (±10.5)	91.4 (±10.2)	<0.001
Fasting insulin (U/l)	14.3 (±9.3)	14.9 (±9.1)	13.8 (±9.5)	0.249
2h OGTT glucose (mg/dl)	120.7 (±29.7)	124.1 (±29.5)	118.2 (±29.6)	<0.05
HOMA-IR	3.30 (±2.2)	3.52 (±2.18)	3.13 (±2.16)	0.063
c-IMT (mm)	0.69 (±0.16)	0.70 (±0.17)	0.67 (±0.15)	0.061
LVMI (g/m^2^)	94.8 (±22.9)	106.5 (±25.5)	86.1 (±16.0)	<0.0001
Smoking habits (Y/N)	83/330	46/134	37/196	<0.01

The data are presented as means ± SD for continuous variables and number (percentages) for dichotomous variables. Comparisons were performed using a general linear model with post hoc Bonferroni correction for multiple comparisons and by the χ^2^ test for categorical variables. *P* values refer to results after analyses with adjustment for age, gender, and BMI. * *P* values refer to results after analyses with adjustment for age and BMI. ** *P* values refer to results after analyses with adjustment for gender and BMI. *** *P* values refer to results after analyses with adjustment for age and gender. Triglycerides and fasting insulin were log transformed for statistical analysis, but values in the table represent back transformation to the original scale. SBP, systolic blood pressure; DBP, diastolic blood pressure; HDL-Chol, high-density lipoprotein cholesterol; LDL-Chol, low-density lipoprotein cholesterol; HOMA-IR, the homeostasis model assessment index of insulin resistance; LVMI, left ventricular mass index; and c-IMT, carotid intima-media thickness.

**Table 2:** Univariate correlations between Mg^2+^ and anthropometric and biochemical variables.

	Unadjusted Correlations Between Mg^2+^ and Metabolic Variables		Age- and Gender-adjusted Correlations Between Mg^2+^ and Metabolic Variables	
Variables	Pearson’s Correlation Coefficient (r)	*P*	Pearson’s Correlation Coefficient (r)	*P*
Age (yr^−1^)	0.082	0.098	0.072	0.146 **
BMI (Kg/m^2^)	−0.144	<0.01	−0.176	<0.001 *
SBP (mg/dl)	0.105	0.032	0.038	0.494
DBP (mmHg)	0.060	0.224	−0.005	0.927
Total cholesterol (mmHg)	0.006	0.908	−0.017	0.754
HDL-Chol (mg/dl)	0.032	0.518	0.124	<0.03
LDL-Chol (mg/dl)	0.029	0.560	−0.004	0.935
Triglycerides (mg/dl)	0.002	0.973	−0.036	0.512
Fasting glucose (mg/dl)	−0.060	0.224	−0.126	<0.03
2h OGTT glucose (mg/dl)	−0.108	<0.03	−0.164	<0.01
FP insulin (mU/mL)	−0.085	0.084	−0.072	0.195
HOMA-IR	−0.102	0.040	−0.098	0.077
c-IMT (mm)	−0.113	<0.03	−0.140	0.010
LVMI (g/m^2^)	−0.122	<0.03	−0.209	<0.001

BMI = body mass index; SBP = systolic blood pressure; DBP = diastolic blood pressure; HDL-Chol = high density lipoprotein; LDL-Chol = low density lipoprotein; FP insulin = fasting plasma insulin; c-IMT = carotid intima-media thickness; LVMI = left ventricular mass index. * *P* values refer to results after analyses with adjustment for age. ** *P* values refer to results after analyses with adjustment for gender.

**Table 3:** Stepwise multivariable regression analysis of the c-IMT index as a dependent variable and different covariates in the whole population.

Dependent Variable c-IMT	Independent Contributors	Standardized Coefficient β	*P*
	AGE	0.440	<0.0001
	BMI	0.225	<0.0001
	Mg^2+^	−0.122	< 0.01
	Gender	0.035	0.505
	SBP	0.065	0.292
**Model**	DBP	−0.075	0.204
	Total- cholesterol	−0.131	0.220
	HDL-Chol	−0.026	0.657
	LDL-Chol	0.182	0.087
	HOMA-IR	−0.036	0.481
	Smoking habits	−0.017	0.706

**Table 4:** Stepwise multivariable regression analysis of LVMI as a dependent variable and different covariates in women (Model 1) and men (Model 2).

Dependent Variable LVMI	Independent Contributors	Standardized Coefficient β	*p*
	AGE	0.244	0.012
	Mg^2+^	−0.177	0.019
	DBP	0.184	0.038
	SBP	0.083	0.383
	BMI	0.150	0.090
Model 1	Total- cholesterol	−0.071	0.710
	HDL-Chol	0.101	0.268
	LDL-Chol	0.091	0.623
	HOMA-IR	0.057	0.512
	Menopause	−0.025	0.787
	Smoking habits	−0.085	0.240
	AGE	0.211	0.019
	Mg^2+^	−0.171	0.038
	HOMA-IR	−0.211	0.041
	BMI	0.221	0.220
Model 2	SBP	0.147	0.176
	DBP	−0.075	0.488
	Total cholesterol	−0.231	0.248
	HDL-Chol	0.070	0.935
	LDL-Chol	0.181	0.354
	Smoking habits	0.034	0.693
